# Recurrent hybridization underlies the evolution of novelty in *Gentiana* (Gentianaceae) in the Qinghai-Tibetan Plateau

**DOI:** 10.1093/aobpla/plaa068

**Published:** 2020-12-02

**Authors:** Peng-Cheng Fu, Alex D Twyford, Shan-Shan Sun, Hong-Yu Wang, Ming-Ze Xia, Cheng-Xi Tan, Xiao-Jun Zhou, Shi-Long Chen

**Affiliations:** 1 School of Life Science, Luoyang Normal University, Luoyang, P.R. China; 2 Ashworth Laboratories, Institute of Evolutionary Biology, The University of Edinburgh, Edinburgh, UK; 3 Royal Botanic Garden Edinburgh, 20A Inverleith Row, Edinburgh, UK; 4 Key Laboratory of Adaptation and Evolution of Plateau Biota, Northwest Institute of Plateau Biology, Chinese Academy of Sciences, Xining, P.R. China; 5 University of Chinese Academy of Sciences, Beijing, P.R. China

**Keywords:** *Gentiana*, hybridization, phylogenetic analysis, postglacial evolution

## Abstract

The Qinghai-Tibetan Plateau (QTP) and adjacent areas are centres of diversity for several alpine groups. Although it is known that the QTP acted as a source area for diversification of the alpine genus *Gentiana*, the evolutionary processes underlying diversity in this genus, especially the formation of narrow endemics, are still poorly understood. Hybridization has been proposed as a driver of plant endemism in the QTP but few cases have been documented with genetic data. Here, we describe a new endemic species in *Gentiana* section *Cruciata* as *G. hoae* sp. nov., and explore its evolutionary history with complete plastid genomes and nuclear ribosomal internal transcribed spacer sequence data. Genetic divergence within *G. hoae* ~3 million years ago was followed by postglacial expansion on the QTP, suggesting Pleistocene glaciations as a key factor shaping the population history of *G. hoae*. Furthermore, a mismatch between plastid and nuclear data suggest that *G. hoae* participated in historical hybridization, while population sequencing show this species continues to hybridize with the co-occurring congener *G. straminea* in three locations. Our results indicate that hybridization may be a common process in the evolution of *Gentiana* and may be widespread among recently diverged taxa of the QTP.

## Teaser

Here, we describe a new species, *Gentiana hoae* from the Qinghai-Tibetan Plateau (QTP), and explore its evolutionary history in this region. Pleistocene glaciations are suggested as a key factor shaping the population history of *G. hoae*. Phylogenetic analysis suggests that *G. hoae* participated in historical hybridization, while population sequencing show this species continues to hybridize with the co-occurring congener *G. straminea* in three locations. Our results indicate that hybridization may be a common process in the evolution of *Gentiana* and may be widespread among recently diverged taxa of the QTP.

## Introduction

The Tibeto-Himalayan region (THR), comprising the Qinghai-Tibetan Plateau (QTP), the Himalayas and the Hengduan Mountains, is one of the major hotspots for cold-adapted lineages (Hagen *et al.* 2019). Numerous studies have associated geological history and climatic change in the THR with inter- and intraspecific genetic divergence (Wen *et al.* 2014; Favre *et al.* 2015; Sun *et al.* 2017; Mosbrugger *et al.* 2018; Muellner-Riehl 2019). Parts of the QTP may have reached 4000 m elevation as early as 40 million years ago (Ma), while the Hengduan Mountains are considered relatively young (Miocene, late Pliocene) (Favre *et al.* 2015; Meng *et al.* 2017) and only reached significant elevation before the Pleistocene (Sun *et al.* 2011). Muellner-Riehl (2019) suggested that the timing, locality and extent of Pleistocene glaciation are key factors underlying the diversity of species and partitioning of genetic variation between populations in the THR. Glacials and interglacials in the THR drastically modified the distribution of species and may have facilitated secondary contact of recently diverged lineages (Wen *et al.* 2014), or caused fragmentation of a species distribution range. Overall, many botanical studies have suggested that Pleistocene climatic fluctuations have promoted diversification of plants in the THR (Qiu *et al.* 2011; Liu *et al.* 2014a; Wen *et al.* 2014; Sun *et al.* 2017; Mosbrugger *et al.* 2018).

The different geological histories of the QTP and the Hengduan Mountains, and in particular the different tempo of mountain uplift, underlie contrasting plant evolutionary patterns in each area (Muellner-Riehl 2019). However, questions remain about the evolutionary patterns of taxa in the junction between these two regions. In the absence of a clear synthesis of the extent of Pleistocene glaciation and climatic history in the THR (Muellner-Riehl 2019), studies of plant evolutionary history may offer indirect insights into past geographic history. The junction between QTP and the Hengduan Mountains such as the Yushu area has a number of endemic species, for example in *Gentiana* (Ho and Pringle 1995) and *Saxifraga* (Pan *et al.* 2001). Phylogeographic studies have shown that the junction area served as a micro-refugium for alpine plants such as *Rhodiola* (Gao *et al.* 2012), *Gentiana* (Lu *et al.* 2015; Fu *et al.* 2018, 2020), *Sibiraea* (Fu *et al.* 2016a) and others (Qiu *et al.* 2011; Liu *et al.* 2012; Muellner-Riehl 2019). Compared with widespread species in the above genera, species endemic to the junction area may shed new light on the Pleistocene history of the alpine flora of the THR.

In addition to geological history and climatic fluctuations, hybridization is another factor that has been proposed to shape genetic variation and species diversity in the THR (Wen *et al.* 2014). Hybridization can be a creative force leading to the introgression of adaptive genetic variation or the generation of new species via hybrid speciation (Abbott *et al.* 2013). On the other hand, hybridization can prevent genetic divergence among taxa and even cause extinction of rare endemics (Buerkle *et al.* 2003). As hybridization is more common in closely related species such as those characterized by recent divergence (Mallet 2007; Nolte and Tautz 2010; Abbott *et al.* 2013), hybridization may be a common process in recent species complexes found in the THR (Liu *et al.* 2014a; Wen *et al.* 2014; Yang *et al.* 2019). To date, hybridization has been reported in the THR in diverse groups such as pine (Ma *et al.* 2006), spruce (Sun *et al.* 2014; Shen *et al.* 2019), *Ostryopsis* (Liu *et al.* 2014b), *Rhododendron* (Yan *et al.* 2017), *Cupressus* (Ma *et al.* 2019) and *Gentiana* (Fu *et al.* 2020).

*Gentiana* (Gentianaceae) is an alpine genus encompassing ca. 360 species (Ho and Liu 2001), with the QTP acting as the primary source region for dispersal to numerous mountain systems across the world (Favre *et al.* 2016). Previous studies in the genus have showed that topographic and climatic change in the THR triggered the recent differentiation of *Gentiana* species (e.g. Zhang *et al.* 2007, 2009; Lu *et al.* 2015; Favre *et al.* 2016; Fu *et al.* 2018, 2020). Although the biogeographic history of *Gentiana* on a global scale is relatively well-understood, the evolutionary processes that have shaped diversity of this genus on the QTP have not been well-characterized. In particular, while most species in the genus are narrow endemics (Ho and Liu 2001), little is known about the population biology of endemic taxa. While population studies have focused on three species with wide ranges (Lu *et al.* 2015; Fu *et al.* 2018, 2020), only one evolutionary study has investigated narrow endemic *Gentiana* species (Zhang *et al.* 2007). Additionally, considering many *Gentiana* species have overlapping distribution areas or are sympatric in the THR (Ho and Pringle 1995; Ho and Liu 2001), and given the relatively weak reproductive barriers among closely related species and the predominance of outcrossing (e.g. Duan *et al.* 2007; Hou *et al.* 2008), hybridization is expected to be common in *Gentiana*. However, few studies have investigated its role in the evolution of this genus. To date, *G. straminea* has been confirmed to hybridize with *G. siphonantha* (Li *et al.* 2008; Hu *et al.* 2016), while another study showed that one clade that includes *G. lawrencei* var. *farreri* originated via hybridization with *G. veitchiorum* (Fu *et al.* 2020).

Here, we firstly describe a new species, *Gentiana hoae* sp. nov., which is an endemic species that belongs to section *Cruciata*. This section is species-rich and has its greatest diversity in the THR (Ho and Liu 2001; Zhang *et al.* 2009). We then investigate the phylogenetic position of *G. hoae* in the context of existing data from diverse species in sect. *Cruciata* by sequencing the plastomes as well as the nuclear ribosomal internal transcribed spacer (nrITS). We then explore the population processes shaping diversity in this endemic species using phylogeographic analysis of two plastid regions and nrITS in dense population-level samples of *G. hoae*. As the phylogenetic analysis revealed a putative hybrid origin of *G. hoae* (see Results), we then investigated whether *G. hoae* continues to hybridize in the area of sympatry between *G. hoae* and *G. straminea* using cloned nrITS data. Overall, we use these results to understand the phylogeographic history of a narrow endemic species, and use this as a case study of the potential role hybridization may play in the generation of novel diversity in the THR.

## Methods

### Study species and plant sampling

*Gentiana* species are classified into 15 sections, and 199 species belonging to 11 sections occur in the THR (Ho and Liu 2001; Yu *et al.* 2020). Section *Cruciata* contains 21 species and these are mainly found across eastern Eurasia (Ho and Liu 2001). Most species within this section are restricted to high-altitude regions in the Asian mountains and only one species is found in Europe (*G. cruciata*). Section *Cruciata* has its greatest species diversity in the THR, where there are 12 endemic species (Ho and Liu 2001; Zhang *et al.* 2009). Cytological investigations determined that seven species are diploids and four are tetraploids (Yuan 1993; Yuan *et al.* 1998; Ho *et al.* 2002). Species in section *Cruciata* are perennials, that are predominantly outcrossing (Ho and Liu 2001), with most visitations from generalist pollinators such as bumblebees (Duan *et al.* 2007).

**Figure 1.**
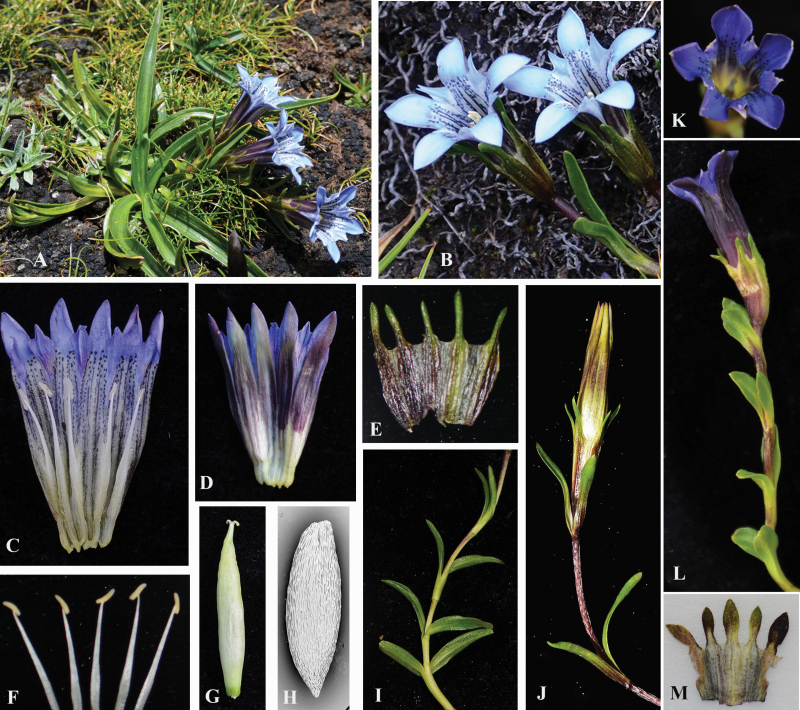
Morphological characteristics of *Gentiana hoae* sp. nov. (A–L) and its close relative *G. lhassica* (K–M). (A) Whole plant in a dry alpine meadow at Yushu; (B) flowers; (C and D) corolla; (E) calyx; (F) stamens; (G) capsule; (H) seed, SEM (200×); (I and J) stem; (K) corolla; (L) stem; (M) calyx. Photographs by Peng-Cheng Fu.

*Gentiana lhassica*, belonging to sect. *Cruciata*, is a gentian species that is morphologically variable. Here, we split the species, with populations with a distinct morphology newly described as *G. hoae* sp. nov. *Gentiana hoae* differs from *G. lhassica* in a number of characteristics including leaf and flower shape. Specifically, *G. hoae* has a stem leaf blade that is lanceolate to linear-lanceolate, calyx lobes narrowly elliptic to linear, corollas that are pale blue and corolla lobes triangular-elliptic (Fig. 1). In contrast, *G. lhassica* has a stem leaf blade that is elliptic-lanceolate to elliptic, calyx lobes narrowly elliptic, corollas that are blue and corolla lobes ovate-orbicular (Fig. 1; Ho and Pringle 1995). The two species have contiguous but distinct distributions: *G. hoae* is distributed in Southwest Qinghai, Northeast Tibet and the western border of Sichuan, and *G. lhassica* is distributed in East Tibet. To quantify how the two species differ in morphology, we measured three key traits (the length/width of the basal leaf blade, stem leaf and calyx lobe) in two natural populations of each species. One population from the type locality of *G. lhassica* (Lhasa, P16, Fu2020007) was included. Sample sizes of individuals ranged from 24 to 62 in different populations (Table 1).

**Table 1.** The key morphological differences between populations of *Gentiana hoae* sp. nov. and its close relative *G. lhassica*. *N* is the number of individuals sampled. Population code: P2, Fu2017046; P3, Fu2017042; P15, Fu2016204; P16, Fu2020007.

		*Gentiana hoae*		*G. lhassica*	
Plant part	Character	P3 (*N* = 24)	P2 (*N* = 62)	P15 (*N* = 51)	P16 (*N* = 50)
Basal leaves	Length/average (mm)	42–93/63.4	48–92/65.9	34–67/50.5	30–65/46.8
	Width/average (mm)	5–9/7.0	4–8/6.0	7–12/9.3	7–14/10.0
	Length-width ratio/average	6–16/9.3	8.6–18.3/11.4	3.8–7.5/5.5	3.1–8.1/4.9
Stem leaves	Length/average (mm)	9–22/15.7	10–27/16.1	7–16/10.2	9–18/13.1
	Width/average (mm)	2–4/2.7	1.5–4.8/2.8	3.5–6.5/4.6	3.5–7/5.4
	Length-width ratio/average	4–8.5/5.9	3.7–8.3/5.9	1.5–3.4/2.2	1.8–3.6/2.4
Calyx lobes	Length/average (mm)	3–8/5.0	3–8/5.0	3.5–7/4.5	3.5–7.5/5.2
	Width/average (mm)	0.5–1/0.8	0.5–1/0.7	1–2.5/1.7	1–3/2.0
	Length-width ratio/average	4.5–9/6.8	5–12/7.8	1.8–4/2.7	1.5–4/2.7

Our sampling, and subsequent genetic analyses, was performed on three distinct data sets. In order to understand the phylogenetic relationship between *G. hoae* and other *Gentiana* species, we sampled this species and its close relatives in sect. *Cruciata* to place the samples in a broader phylogenetic context. In order to explore the evolutionary history of *G. hoae*, we collected six populations totalling 84 individuals throughout the QTP. For investigating hybridization between *G. hoae* and congeneric species, we also collected eight populations of *G. straminea* from the area overlapping with the distribution of *G. hoae* (Table 2). *Gentiana straminea* is one of the most common and dominant species of sect. *Cruciata* in the THR and has a sympatric distribution with *G. hoae*. One population of *G. lhassica* with 14 individuals was collected as an allopatric reference population for comparison. For small populations (<100 individuals; *N*_pop_ = 4), 25–50 % of plants were sampled. For large populations (>100 individuals; *N*_pop_ = 10), 10–20 mature plants were randomly sampled. Young leaves were dried in silica gel. Voucher specimens were deposited in the herbarium of the School of Life Science, Luoyang Normal University.

**Table 2.** Summary genetic statistics for *Gentianan hoae* and its closely related species. P., population code; No., sample size; *h*, gene diversity; π, nucleotide diversity. Abbreviation after localities indicates provinces as follows: QH, Qinghai; T, Tibet.

					Chloroplast			nrITS		
P.	Voucher ref.	Locality	Latitude and longitude	No.	Haplotype composition	*h*	π (10^−3^)	Haplotype composition	*h*	π (10^−3^)
*G. hoae*										
P1	Fu2017034	Chenduo, QH	N33°07′/E97°27′	12	Hc1(9), Hc2(3)	0.409	0.842	H2(1), H3(7), H4(4)	0.591	1.054
P2	Fu2017042	Yushu, QH	N33°06′/E96°45′	19	Hc1(16), Hc4(1), Hc9(1)	0.216	0.457	H2(1), H3(9), H4(9)	0.579	1.022
P3	Fu2017046	Yushu, QH	N32°46′/E97°12′	20	Hc1(17), Hc8(1), Hc9(2)	0.279	0.747	H2(1), H3(12), H4(7)	0.542	0.937
P4	Fu2017062	Yushu, QH	N32°53′/E96°41′	3	Hc1(1), Hc9(2)	0.667	2.058	H2(2), H3(1)	0.667	1.079
P5	Fu2017072	Nangqian, QH	N31°58′/E96°30′	16	Hc1(10), Hc6(2), Hc7(4)	0.567	0.926	H1(12), H3(4)	0.325	0.525
P6	Fu2017135	Changdu, T	N31°21′/E97°40′	15	Hc1(11), Hc3(3), Hc5(1)	0.448	0.490	H3(4), H5(1), H6(1), H7(1), H8(3), H9(1), H10(2), H11(1), H12(1)	0.905	3.976
*G. straminea*										
P7	Fu2017027	Chenduo, QH	N33°07′/E97°27′	12	Hc1(10), Hc2(1), Hc6(1), Hc14(1)	0.423	0.871	S1(11), S2(1)	0.154	0.248
P8	Fu2017040	Zhiduo, QH	N33°33′/E96°03′	10	Hc8(6), Hc9(1), Hc10(1), Hc15(2)	0.644	1.462	S1(6), S2(1), S3(1), S4(1), S5(1)	0.667	1.649
P9	Fu2017049	Yushu, QH	N33°06′/E96°45′	8	Hc1(5), Hc3(1), Hc5(1), Hc8(1)	0.643	1.028	S1(7), S2(1)	0.250	0.403
P10	Fu2017066	Yushu, QH	N32°46′/E97°12′	4	Hc1(4)	0.000	0.000	S1(4)	0.000	0.000
P11	Fu2017080	Nangqian, QH	N31°58′/E96°30′	8	Hc1(1), Hc2(4), Hc7(1), Hc12(1), Hc15(1)	0.786	2.019	S1(8)	0.000	0.000
P12	Fu2017093	Dingqing, T	N31°20′/E95°43′	10	Hc1(4), Hc9(1), Hc15(5)	0.644	2.215	S1(6), S6(4)	0.533	0.860
P13	Fu2017116	Changdu, T	N31°24′/E97°20′	10	Hc1(1), Hc6(1), Hc9(1), Hc11(6), Hc13(1)	0.667	1.939	S1(9), S7(1)	0.200	0.323
P14	Fu2018142	Chayu, T	N29°19′/E97°03′	13	Hc6(13)	0.000	0.000	S1(11), S8(2)	0.282	0.455
Hybrids										
Hyb1	Fu2017043	Yushu, QH	N33°06′/E96°45′	1	Hc16(1)					
Hyb2	Fu2017051	Yushu, QH	N32°46′/E97°12′	3	Hc1(1), Hc3(1), Hc8(1)					
Hyb3	Fu2017136	Changdu, T	N31°21′/E97°40′	1	Hc1(1)					
*G. lhassica*										
P15	Fu2016204	Mozhugongka, T	N29°49′/E92°21′	14	Hc17(14)	0.000	0.000	L1(14)	0.000	0.000

### Phylogenetic analysis in section *Cruciata*

#### Molecular protocols.

We newly sequenced the plastome of *G. lhassica* and subsequently reconstructed phylogenetic relationships in sect. *Cruciata* with 12 previously published plastomes (including *G. hoae*). Total genomic DNA isolation, DNA fragmentation and sequencing library construction followed the process described in Fu *et al.* (2016b). The fragmented genomic DNA was sequenced using the Illumina HiSeq 4000 platform (Novogene, Tianjing, China), generating 150-bp paired-end reads. The plastome was assembled *de novo* using NOVOPlasty 2.6.1 (Dierckxsens *et al.* 2016) and annotated with PGA (Qu *et al.* 2019) using the default parameters. The newly sequenced plastome was deposited in GenBank (MT982398).

For assessing the phylogenetic position of *G. hoae* in sect. *Cruciata* using nuclear data, nrITS (Taberlet *et al.* 1991) was amplified in *G. hoae* and *G. lhassica*, respectively. Total genomic DNA was extracted with a Dzup plant genomic DNA extraction kit (Sangon, Shanghai, China). The PCRs were performed in 20 μL volumes containing 1× PCR Buffer, 1.5 mM MgCl_2_, 0.3 mM of each dNTP, 0.3 mM of each forward and reverse primer, 1 unit of *Taq* DNA polymerase (Takara, Dalian, China) and 10–40 ng template DNA. The PCR cycling profile included an initial step of 5 min at 95 °C followed by 33 cycles of denaturation at 95 °C for 50 s, 50 s of annealing at 55 °C and 30 s at 72 °C, with a final extension at 72 °C for 6 min. PCR products were sequenced on an ABI 3730 xl automated capillary sequencer (Applied Biosystems, Foster City, CA, USA) with BigDye v3.1 (Applied Biosystems).

#### Phylogenetic analysis.

In addition to the newly sequenced plastomes, another 12 plastomes in sect. *Cruciata* were retrieved from GenBank (see Results) to place the relationship of our study species in a broader phylogenetic context. Sequences of all protein-coding genes were extracted from each plastome in PhyloSuite (Zhang *et al.* 2020) and aligned using MAFFT (Katoh *et al.* 2002). A protein-coding matrix was constructed after excluding genes that were absent in some species, or that showed high-sequence variability that made alignment difficult. The new nrITS sequences, along with the available data in GenBank were aligned with GENEIOUS PRO 3.5.6 (Kearse *et al.* 2012). Phylogenetic relationships were analysed using maximum likelihood (ML) and Bayesian inference (BI). The best-fitting models of sequence evolution were selected in ModelFinder (Kalyaanamoorthy *et al.* 2017) based upon the AIC and BIC criterion. The ML analyses were conducted in IQ-TREE (Nguyen *et al.* 2015) with the robustness tested with 1000 bootstrap replicates. The BI analyses were performed with MrBayes 3.2.6 (Ronquist *et al.* 2012) implemented in the PhyloSuite platform (Zhang *et al.* 2020). We performed two simultaneous runs from random starting trees, with four coupled incrementally heated Markov chains each. We ran the chains for 10 million generations and sampled every 1000th generation. The initial 10 % of sampled data were discarded as burn-in.

### Phylogeographic analysis in *G. hoae*

#### Molecular protocols.

To investigate population genetic structure of *G. hoae*, nrITS (Taberlet *et al.* 1991) and two intergenic plastid spacer regions, *trnS*(GCU)-*trnG*(UCC) (Hamilton 1999) and *rpl32-trnL,* which is highly variable in *Gentiana* (Sun *et al.* 2018), were amplified in all individuals with PCRs and profiles as above. The following primer sequences were used to amplify *rpl32-trnL*, F: CAAACRAATGAGCACAATAAAA; R: CCTAAGAGCAGCGTGTCTACCA. PCR products were sequenced on an ABI 3730 xl automated capillary sequencer (Applied Biosystems).

#### Phylogeographic analysis.

Sequences were aligned and edited with GENEIOUS PRO 3.5.6 (Kearse *et al.* 2012). Haplotypes were identified in DnaSP 5.1 (Librado and Rozas 2009) and new sequences were deposited in GenBank (plastid: MN399866–MN399871; nrITS: MN400709–MN400720). Gene diversity (*h*) and nucleotide diversity (π) indices were calculated in ARLEQUIN 3.5 (Excoffier and Lischer 2010). To estimate differentiation among populations, the coefficients of differentiation *G*_ST_ and *N*_ST_ were calculated using the software PERMUT (Pons and Petit 1996). Hierarchical analysis of molecular variance (AMOVA; Excoffier *et al.* 1992) was used to further quantify genetic differentiation of *G. hoae* using the software ARLEQUIN, with 1000 permutations. To explore demographic history such as potential population growth or expansion we calculated Fu’s *Fs* (Fu 1997) and Tajima’s *D* (Tajima 1989) using 10 000 simulations in ARLEQUIN.

To estimate the phylogenetic relationship among haplotypes, ML analyses were conducted in IQ-TREE (Nguyen *et al.* 2015) using the best-fitting model estimated in ModelFinder (Kalyaanamoorthy *et al.* 2017), which was TPM3+F+I (plastid data) or GTR+F+I (nrITS data). The robustness of the ML trees was tested with 1000 bootstrap replicates. A median-joining (MJ) haplotype network was calculated in NETWORK 4.6 (Bandelt *et al.* 1999).

We estimated the divergence times with a Bayesian method implemented in BEAST 2.4.6 (Bouckaert *et al.* 2014). We only estimated divergence times for the nrITS sequence data and not the plastid sequences, as the nrITS showed sufficient sequence variation without extensive haplotypes sharing (see Results). We used the GTR substitution model, the Yule model and lognormal clock model (Drummond *et al.* 2006). To calibrate divergence times, we constrained the node of sect. *Cruciata* with a date of 5.0 Ma based on the well-documented seed fossil assigned to this section (Mai and Walther 1988). We used a lognormal prior with a mean of 0.7, and a standard deviation of 1.0 (Pirie *et al.* 2015; Favre *et al.* 2016). We ran three independent MCMC chains with 10 000 000 generations, sampling every 1000th generation and discarding the initial 10 % as burn-in. Convergence was confirmed in TRACER 1.5 (http://tree.bio.ed.ac.uk/software/tracer/) and judged by effective sample size values (>200). Trees were summarized using TreeAnnotator 1.7.5 (Drummond *et al.* 2012).

### Investigation of hybridization between *G. hoae* and *G. straminea*

#### Molecular protocols.

For studying potential hybridization between *G. hoae* and *G. straminea*, *trnS*(GCU)-*trnG*(UCC) (Hamilton 1999), *rpl32-trnL* and nrITS (Taberlet *et al.* 1991) were amplified in *G. straminea* individuals and five putative hybrid individuals identified based on intermediate morphologies, with PCRs and profiles as above. PCR products were sequenced on an ABI 3730 xl automated capillary sequencer (Applied Biosystems). For five putative hybrid individuals showing double peaks in the electropherograms, PCR products of the nrITS were purified by an eZNA DNA Gel Extraction Kit (Omega Bio-Tek, Guangzhou, China). After the concentration was measured using a NanoDrop 2000c Spectrophotometer (Thermo Scientific), the purified PCR products were ligated into pMD18-T vectors (Takara, Dalian, China), which were then transformed into Trans5α Chemically Competent Cells (TransGen, Beijing, China). Positive clones were tested in a 20-μL PCR volume containing 10–100 ng template DNA, 1× PCR Buffer, 1.5 mM MgCl_2_, 0.2 mM of each dNTP, 0.2 mM of M13F/R and 1 unit of *Taq* DNA polymerase (Takara, Dalian, China). PCR was performed with the following program: an initial step of 5 min at 95 °C followed by 20 cycles of 30 s at 95 °C, 1 min at 53 °C and 30 s at 72 °C, followed by a final extension step at 72 °C for 6 min. For putative hybrids, six clones were sequenced for each individual except one individual where 10 clones were sequenced. The positive clones were sequenced with M13 universal primers.

#### Sequence analysis.

Sequences were aligned and edited with GENEIOUS PRO 3.5.6 (Kearse *et al.* 2012). Haplotypes were identified in DnaSP 5.1 (Librado and Rozas 2009) and new sequences were deposited in GenBank (plastid: MN399872–MN399877; nrITS: MN400721–MN400754, MN400985–MN400992).

## Results

### Morphological differentiation between *G. hoae* and *G. lhassica* and phylogenetic tree of section *Cruciata*

**Figure 2.**
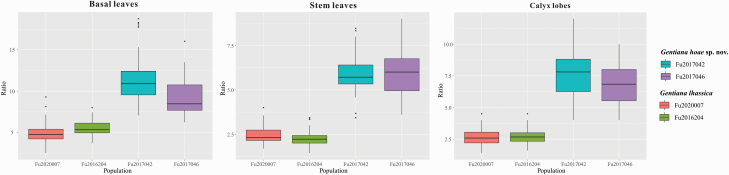
Boxplots of three key morphological characteristic (basal leaves, stem leaves and calyx lobes) in populations of *Gentiana hoae* sp. nov. and its close relative *G. lhassica*.

A total of 86 and 101 individuals of *G. hoae* and *G. lhasscia*, respectively, were measured for three key traits in natural populations. The measurements showed that the species differ in leaf blade, stem leaf and calyx lobe morphologies (Table 1; Fig. 2). For example, the average length-width ratio of stem leaves is 5.9 in *G. hoae*, while ranging from 2.2 to 2.4 in *G. lhasscia*.

**Figure 3.**
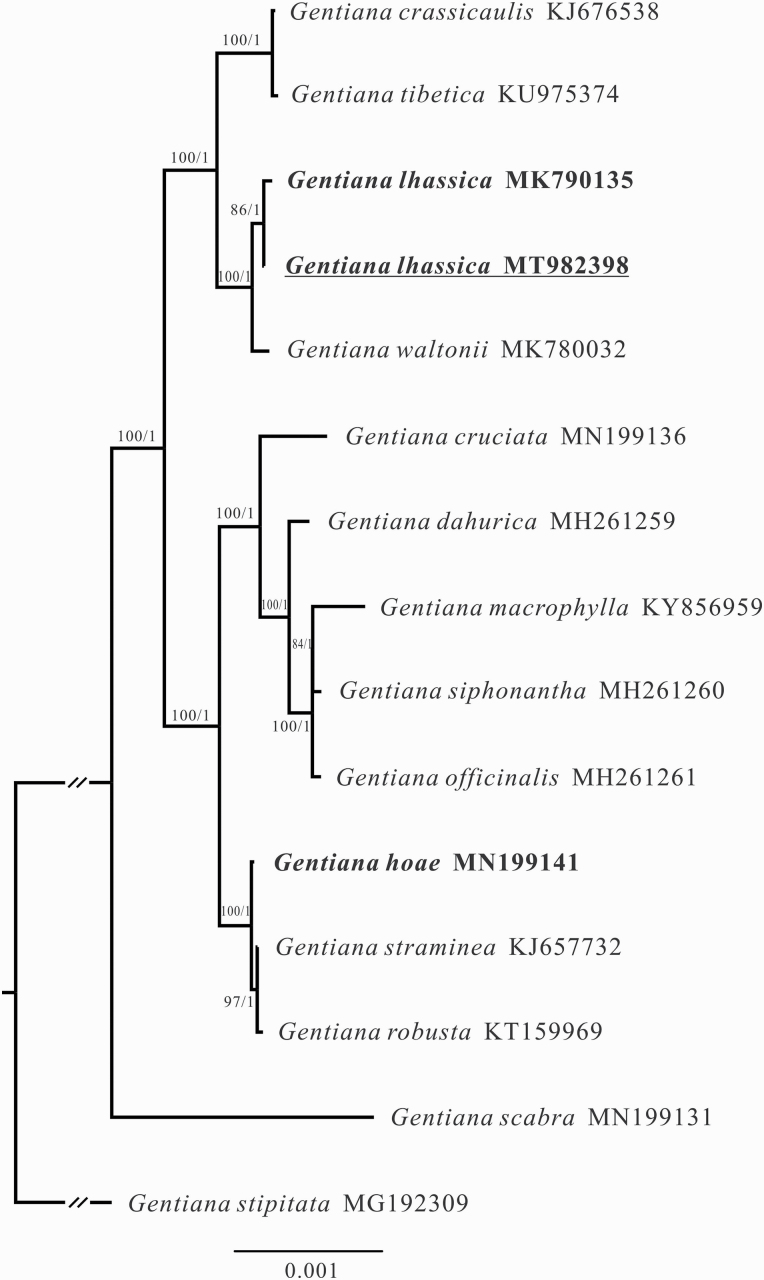
Phylogenetic tree of *Gentiana* section *Cruciata* based on complete plastome sequences. Bootstrap support values obtained from ML analyses and Bayesian posterior probabilities are presented at nodes. The double slash (//) symbolizes an artificial shortening of this branch for visualisation.

The newly sequenced plastome of *G. lhassica* was 148 653 bp in length, and had a very similar structure and gene composition to other sect. *Cruciata* plastomes. Together with previously published data, plastome sequences for 12 out of 21 species in *G*. sect. *Cruciata* were used in the phylogenetic analysis. All sampled species of *G*. sect. *Cruciata* formed a well-supported monophyletic clade (1.00 Bayesian posterior probability, PP; 100 % bootstrap support, BS). Section *Cruciata* has two well-supported clades (1.00, PP; 100 %, BS) where *G. hoae* and *G. lhassica* occurred, respectively (Fig. 3). *Gentiana hoae* clustered with *G. straminea* and *G. robusta*, while *G. lhassica* clustered with *G. waltonii*.

**Figure 4.**
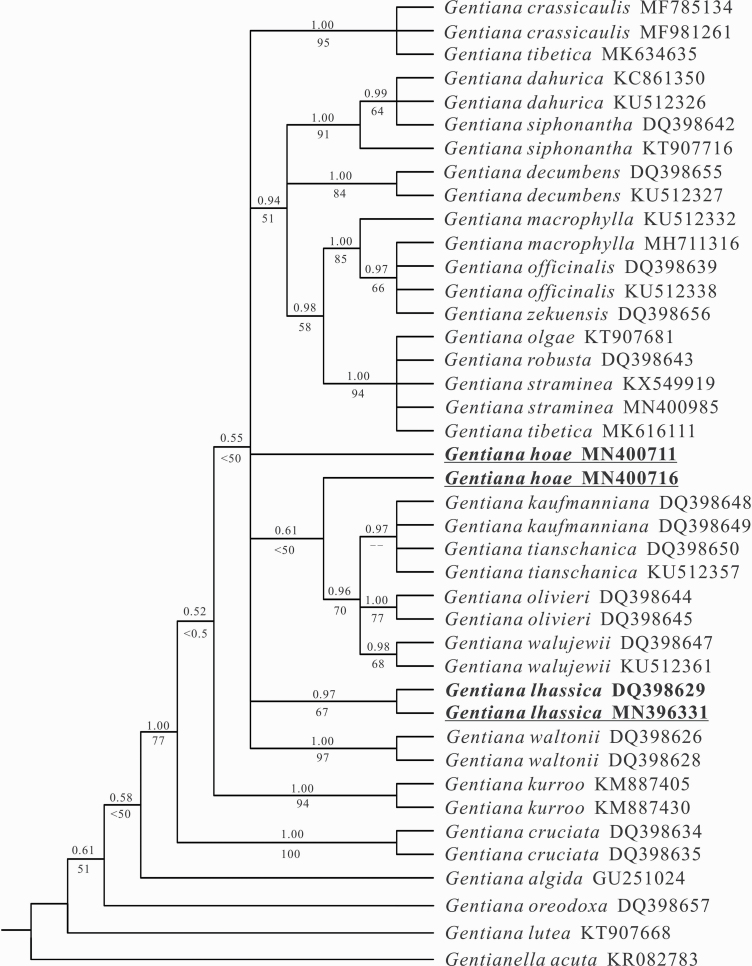
Bayesian inference topology of *Gentiana* section *Cruciata* from internal transcribed spacer regions of the nuclear ribosomal (nrITS) data set. Bayesian posterior probabilities are placed above branches, and BS values obtained from ML analyses are presented below branches.

The nrITS sequences, including the two newly sequenced, represented 18 out of 21 species in *G*. sect. *Cruciata*. The length of the aligned nrITS sequences were 595 bp, in which 44 nucleotide substitutions and five indels were detected. One consistent nucleotide difference was detected between *G. hoae* and *G. lhassica*. All sampled species of *G*. sect. *Cruciata* formed a well-supported monophyletic clade (PP 1.00, BS 77 %). The BI topology of sect. *Cruciata* showed highly supported intra-sectional structure with most nodes having a PP above 0.95, but with relatively low BS. One sequence of *G. hoae* clustered in one clade with weak support (PP 0.61, BS < 50 %) and the other sequence was placed on its own. Two sequences of *G. lhassica* clustered as a moderately supported clade (PP 0.97, BS 67 %) (Fig. 4).

### Genetic structure and evolutionary history of *G. hoae*

**Figure 5.**
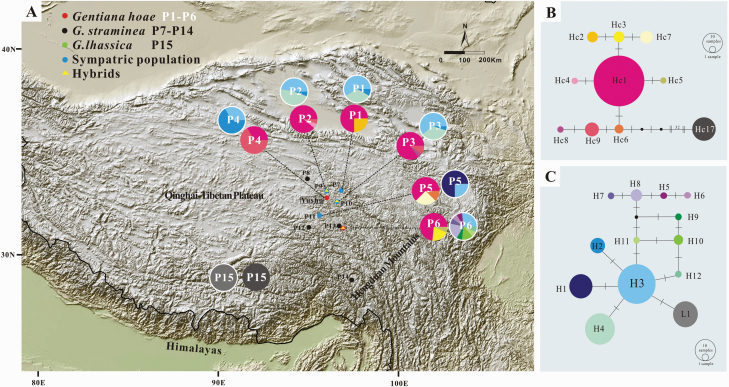
Geographical distribution and networks of plastid haplotypes (*trnS*-*trnG* and *rpl32-trnL* loci) and the internal transcribed spacer region in *Gentiana hoae* sp. nov. and closely related species. (A) Geographical distribution of the plastid haplotypes (inner pie charts) and ribotypes (outer pie charts, with white circles) across sampled populations. Pie charts display haplotype/ribotype frequencies in each locality. Map came from the Institute for Planets. (B) Network of the plastid haplotypes. (C) Network of the nrITS ribotypes. The relative sizes of the circles in the network are proportional to haplotype/ribotype frequencies. One short dash represents one nucleotide variation and black dots represent missing haplotypes.

The aligned sequences of the *trnS*(GCU)-*trnG*(UCC) and *trnL-rpl32* were 534 bp and 440 bp in length, respectively. The two plastid fragments were concatenated to perform the following haplotypic analyses. The plastid data set included four base substitutions and four indels **[see**  **Supporting Information—Appendix S1****]** that identified nine haplotypes (Hc1–Hc9) in *G. hoae* (Table 2; Fig. 5A). All individuals of *G. lhassica* have a single haplotype (Hc17). One haplotype (Hc1) was shared in all populations of *G. hoae* and seven were exclusive to one population. The *G*_ST_ and *N*_ST_ were 0.162 and 0.268 (*P* < 0.05), respectively, suggesting significant phylogeographic structure in this species. Analysis of molecular variance revealed that most genetic variation occurred within populations (81.53 %) rather than among populations (18.47 %) (Table 3). The network of plastid haplotypes showed that the common haplotype Hc1 was central in *G. hoae* (Fig. 5B), suggesting population expansion occurred recently. Neutrality test showed that Tajima’s *D* was −0.653 (*P* = 0.277) and Fu’ *Fs* was −4.151 (*P* = 0.02), thus indicated a recent population expansion.

**Table 3.** Analyses of molecular variance in *Gentianan hoae* based on the chloroplast and the internal transcribed spacer.

Source of variation	Degrees of freedom	Sum of squares	Variance components	Percentage of variation
Chloroplast				
Among populations	5	7.120	0.0792 Va	18.47
Within populations	79	27.261	0.3495 Vb	81.53
nrITS				
Among populations	5	24.505	0.3249 Va	42.26
Within populations	79	35.072	0.4440 Vb	57.74

**Figure 6.**
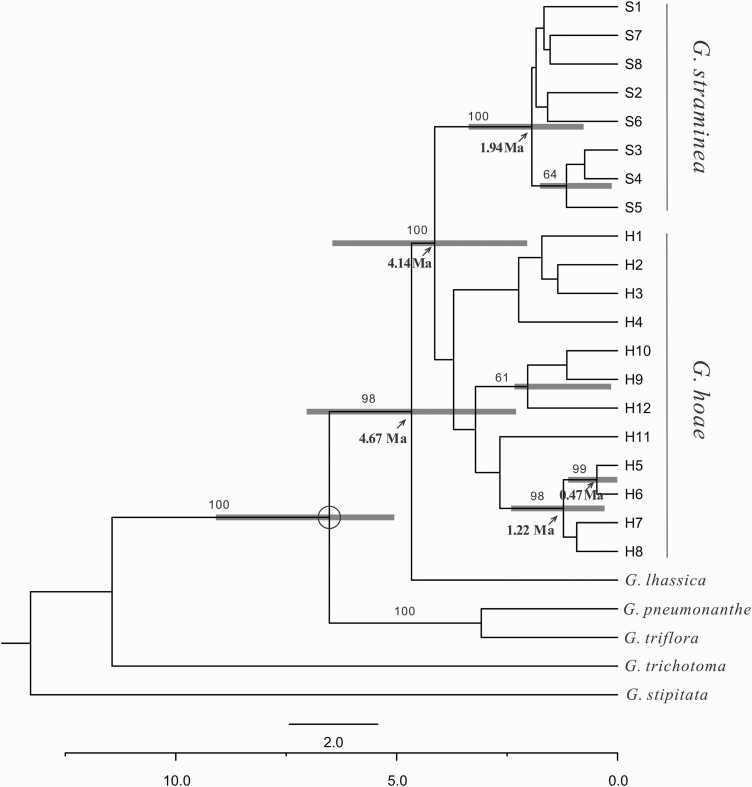
Majority rule consensus phylogenetic tree and divergence times based on *Gentiana hoae* sp. nov. and closely related species, based on the BI. The tree is based on an alignment of the internal transcribed spacer region. Fossil seeds of *Gentiana* were used for setting temporal constraints and indicated by a circle around the calibration node. Numbers on the branches indicate the Bayesian PP (only values >70 % are indicated). Node ages represent mean ages (Ma) and bars show the 95 % HPD.

The alignment of the nrITS region was 621 bp in length. The nrITS data set consisted of eight base substitutions and two indels **[see**  **Supporting Information—Appendix S2****]** that identified 12 ribotypes (H1–H12) in *G. hoae*. All populations of *G. hoae* had more than one ribotype and only three ribotypes (H2–H4) were shared by more than one population (Fig. 5A). The *G*_ST_ and *N*_ST_ was 0.249 and 0.430 (*P* < 0.05), respectively, suggesting significant phylogeographic structure. Analysis of molecular variance revealed that the percentage of variation within populations (57.74 %) was higher than that among populations (42.26 %) (Table 3), though less variation was partitioned within populations than the plastid data. All ribotypes of *G. hoae* clustered in a single clade in the ML tree but with low BS for specific nodes **[see**  **Supporting Information—Appendix S3****]**. The relationship among the ribotypes from the network analysis was consistent with the ML tree, and indicated that ribotype H3 is central in *G. hoae* (Fig. 5C). In addition, network analysis indicated that the ribotype in *G. lhassica* was closely related to *G. hoae* and only differed by one nucleotide substitution from ribotype H3. Neutrality tests showed that Tajima’s *D* was −0.849 (*P* = 0.217) and Fu’ *Fs* was −4.440 (*P* = 0.04), suggesting recent expansion. The divergence between *G. lhassica* and the remaining two species, based on the dated molecular phylogenetic analysis, occurred ~4.67 Ma (95 % highest posterior density, HPD: 2.30–7.04 Ma). *Gentiana hoae* appears to have diverged ~4.14 Ma (HPD: 2.05–6.46 Ma) from *G. straminea*. The intraspecific divergence within *G. hoae* and *G. straminea* mostly occurred within 3 Ma and 2 Ma, respectively (Fig. 6).

### Natural hybridization between *G. hoae* and *G. straminea*

A total of 14 plastid haplotypes were identified in *G. straminea* (Hc1–Hc3 and Hc5–Hc15). Among the four haplotypes (Hc1, Hc3, Hc8 and Hc16) identified in the putative hybrids between *G. hoae* and *G. straminea* based on morphology, three were shared with both potential parents. Among the haplotypes identified in this study, one was exclusive to *G. hoae* (Hc4), six were exclusive to *G. straminea* (Hc10–Hc15), and one was exclusive to putative hybrids (Hc16; Table 2; **see**  **Supporting Information—Appendix S4**).

The nrITS analysis revealed eight new ribotypes (S1–S8) in *G. straminea* with none of these shared with *G. hoae*. The alignment of nrITS sequences was 621 bp in length and consisted of 19 base substitutions and three indels **[see**  **Supporting Information—Appendix S2****]**. Phylogenetic analysis based on the ribotypes showed that *G. straminea*, *G. hoae* and *G. lhassica* were in distinct clades (Fig. 6). Among the variable positions in nrITS, six positions distinguish *G. hoae* and *G. straminea* (Table 4). After alignment, the 34 cloned nrITS sequences included 35 base substitutions and two indels **[see**  **Supporting Information—Appendix S2****]** that identified 33 ribotypes in which one (H3) was shared with *G. hoae* and one (S1) was shared with *G. straminea*. Focusing on the six positions that distinguish *G. hoae* and *G. straminea*, each hybrid individual combined diagnostic sites from both species at each of the six sites (Table 4). In addition to species-specific variation in *G. hoae* and *G. straminea*, recombinant haplotypes were also detected within each hybrid individual (Table 4). The result that species-specific ribotypes are heterozygous in the putative hybrids supports them being early generation hybrids such as F1s.

**Table 4.** Haplotyic comparison of the internal transcribed spacer region of the nuclear ribosomal DNA (nrITS) in *Gentiana hoae* and its hybrids with *G. straminea*. Each sample is listed in turn, along with the variable nucleotide sites. Dots represent the first nucleotide(s) in each column.

	Nucleotides					
Sample	5	14	15	22	31	33
*G. hoae*	C	C	–	T	C	C
*G. straminea*	A	T	TGA	G	–	T
Hyb1	.	.	.	.	.	.
	.	.	.	G	.	.
	.	.	.	.	–	T
	A	T	TGA	G	–	T
Hyb2_1	.	.	.	.	.	.
	.	.	.	.	–	T
	A	T	TGA	G	–	T
	A	T	TGA	.	.	.
Hyb2_2	.	.	.	.	.	.
	.	.	.	G	.	.
	A	T	TGA	G	–	T
	A	T	TGA	G	.	.
Hyb2_3	.	.	.	.	.	.
	.	.	.	G	–	T
	.	.	.	.	–	T
	A	.	.	.	–	T
	A	T	TGA	G	–	T
Hyb3	.	.	.	.	.	.
	.	.	.	G	–	T
	A	T	TGA	G	–	T
	A	T	TGA	G	.	.

## Discussion

Our study shows that *G. hoae* is a new endemic species in *Gentiana* that is morphologically and genetically distinct from other congeners. Phylogenetic evidence suggests this species may have participated in historical hybridization, and continues to hybridize with related species in separate locations. These results indicate that hybridization may be a common process in the evolution of *Gentiana*. In addition, phylogeographic analysis suggests population fragmentation followed by range expansion after Pleistocene glaciations underlie divergence and the subsequent spread of *G. hoae* in the THR.

### Phylogenetic position of *G*. *hoae* and its role in hybridization

Despite *G. hoae* and the congeneric taxon *G. lhassica* being similar, there are notable morphological differences. Compared with the type (K, K000857086) and observations from natural populations of *G. lhassica*, which were collected from the east of Tibet, *G. hoae* has narrower basal leaf blades, stem leaves, calyx and corolla lobes, and a lighter corolla colour (Table 1; Figs 1 and 2). Besides these morphological differences, *G. hoae* has a distinct distribution range adjacent to *G. lhasscia*. *Gentiana hoae* is distributed in Southwest Qinghai, Northeast Tibet and west border of Sichuan to Tibet, and *G. lhassica* is distributed in East Tibet. The habitat of *G. hoae* is very similar to that of *G. lhassica*, with both growing in alpine meadows or scrub. Due to the recent rapid divergence in sect. *Cruciata* (Zhang *et al.* 2009; Favre *et al.* 2016), some gentians in this section differ in relatively few traits, for example, *G. crassicaulis* and *G. tibetica* only differ in corolla length (2–2.2 cm vs. 2.6–3.2 cm) (Ho and Pringle 1995). However, these two species are distinguishable with molecular data, thus providing genetic support for these narrowly divided species (Zhang *et al.* 2006). In general, many *Gentiana* sections have undergone recent speciation and have species that differ by few traits. For instance, in sect. *Kudoa*, *G. dolichocalyx* is considered a distinct species based on genetic data (Fu *et al.* 2020) but only differs morphologically from *G. lawrencei* var. *farreri* by its longer calyx lobes (Ho and Pringle 1995). Based upon phylogenetic analyses, *G. hoae* may be the new species mentioned in Zhang *et al.* (2009) where only plastid fragment data were used. However, no further work has been done on this species and there is no formal taxonomic description. In the future, it would be instructive to perform more intensive sampling of *G. hoae* and its congeners to better understand the nature of species differences of these complex taxa in the QTP.

While it is difficult to trace the evolutionary origins of species in recent radiations, our phylogenetic and phylogeographic analyses allow us to consider possible mechanisms underlying speciation. Molecular phylogenetic analyses based on whole-plastid genome data confirmed that *G. hoae* contains a plastid more closely related to *G. straminea* and *G. robusta* than *G. lhassica* (Fig. 3). The phylogenetic topology in this study was consistent with Zhou *et al.* (2018), but differs from Zhang *et al.* (2009), in which the new species was more closely related to *G. tibetica*. Since *G. tibetica*, which is tetraploid (Yuan *et al.* 1998), has significantly different morphological characters to *G. hoae*, along with many distinguishing sites in the plastome data set, we believed that *G. hoae* and *G. tibetica* are not close relatives. All studies to date have confirmed that *G. lhssica* clusters with *G. waltonii*, with both species’ ranges limited to East Tibet and possessing similar morphological characters. While the lack of phylogenetic resolution in nrITS data prevents us from precisely assessing the relationship of *G. hoae* with closely related species in sect. *Cruciata*, only one nucleotide difference between *G. hoae* and *G. lhassica* in nrITS implies that they are closely related (Fig. 5C). The unsupported topology from nrITS, which has been found in previous studies of sect. *Cruciata*, as well as other groups within *Gentiana* (Yuan and Küpfer 1997; Favre *et al.* 2016; Liu *et al.* 2016), shows recent rapid species diversification in this section (Zhang *et al.* 2009). Considering the results of phylogenetic analyses based on plastid and nrITS data sets, it is likely that *G. hoae* has participated in, and is potentially a product of, one (or more) historical hybridization events. This may have resulted in the capture of a chloroplast haplotype from a relative of *G. straminea* or *G. robusta* in a genetic background more similar to *G. lhassica*. Because not all sect. *Cruciata* species were sampled in our analyses, we cannot rule out that species not included in this study may have participated in this hybridization event, or perhaps this involved an extinct relative. Such ‘genetic ghosts’ participate in hybrid speciation in *Senecio* (Pelser *et al.* 2012) and spruce (Ru *et al.* 2018). Furthermore, since there is consistent morphology in all populations of *G. hoae*, and as *G. hoae* does not have elevated heterozygosity at nrITS as observed in recent hybrids, we consider *G. hoae* is most likely a stable species that may have participated in historical hybridization. Although phylogenetic analysis based on nrITS in section *Cruciata* cannot distinguish *G. hoae* from *G. lhassica*, phylogenetic and network analysis of natural populations suggest that they are distinct, with high support. This highlights the value of population-level analysis for understanding evolutionary relationships in recently diverged groups.

### Genetic divergence and evolutionary history of *G. hoae*

Narrow endemics often harbour low genetic diversity, although high diversity has been observed in some endemics such as those from the Mediterranean mountains (Jiménez-Mejías *et al.* 2015). Compared with species that have wide distribution ranges in the THR, for example species in *Rhodiola* (Gao *et al.* 2012), *Saxifraga* (Li *et al.* 2018a), *Eriophyton* and *Chionochari*s (Luo *et al.* 2016), which all had high genetic diversity, we observed lower genetic diversity in *G. hoae*. However, we did not observe a strong bottleneck effect. Instead, we found that many haplotypes or ribotypes are exclusive to a single population, and that most variation occurred within rather than among populations, indicating some population isolation.

Narrow endemics of the THR may be characterized by a different evolutionary history to widespread species, which could retreat to warm southern refugia during periods of glaciation. Narrow endemics that originate before the Pleistocene may survive *in situ* during glacial periods, immigrate to suitable habits or become extinct. However, many narrow endemic species would be expected to originate during the Pleistocene, where allopatric speciation could occur due to habitat fragmentation in response to glaciation (Hewitt 2004), or alternatively speciation may occur following hybridization after secondary contact (Liu *et al.* 2012; Wen *et al.* 2014). Our estimated timing of divergence among *G. hoae*, *G. lhassica* and *G. straminea* was inferred to be around 2.05–7.04 Ma, consistent with Favre *et al.* (2016) but earlier than Zhang *et al.* (2009). As *G. hoae* is of potential hybrid origin, this divergence time based on a single locus (nrITS) may not accurately reflect the time of origin of the species; however, it is indicative of the general time of divergence in this group. Moreover, the divergence within *G. hoae* occurred around 3.0 Ma, indicating that *G. hoae* may have originated before the Pleistocene and have survived through Pleistocene glaciation in local refugia. Evolutionary studies of narrow endemics in the THR, for instance the alpine taxa *Rhodiola* (Li *et al.* 2018b), have showed similar patterns of local survival through glacial periods. Previous phylogeographic studies have indicated that the Yushu area, within the distribution range of *G. hoae*, has acted as a refugium for several alpine plants (Gao *et al.* 2012; Fu *et al.* 2016a) including *Gentiana* species (Lu *et al.* 2015; Fu *et al.* 2018, 2020). Although several cold-adapted species were found to survive in the central QTP during glacial periods (Liu *et al.* 2012; Muellner-Riehl 2019), the identification of such small refugia requires more extensive nuclear genomic sequencing (Liu *et al.* 2014a), as well as additional fossil evidence to more accurately date phylogenies.

Plastid and nuclear data consistently suggest that *G. hoae* experienced recent population size expansion. Rather than expanding from the refugium in southern Hengduan Mountains (Liu *et al.* 2012; Qiu *et al.* 2011; Muellner-Riehl 2019), *G. hoae* is likely to have experienced local expansion on the QTP platform, as has also been detected in a number of alpine plants, e.g. *Gentiana* (Fu *et al.* 2018) and *Rhodiola* (Li *et al.* 2018b). In addition, local expansion on the THR is likely, considering that the land surface area increases with increasing altitude and range sizes of montane plants increase, rather than decrease, under climate warming (Elsen and Tingley 2015; Liang *et al.* 2018). The distributional ranges of some cold-tolerant conifers (Liu *et al.* 2014a) and subnival herbs (Luo *et al.* 2016) have also expanded or stabilized during glacial cycles that have affected the THR. The colonization of novel habitats may be promoted by potential hybridization in *Gentiana*, as hybridization promotes the evolution of biological novelty (Abbott *et al.* 2013) and the colonization of novel habitats (Rieseberg *et al.* 2003, 2007).

### Natural hybridization between *G. hoae* and *G. straminea*

In this study, hybridization is supported by the presence of individuals combining nrITS copies from *G. hoae* and *G. straminea* at locations where the species grow sympatrically. *Gentiana hoae* is not the first species that has been found to hybridize with *G. straminea,* which is a widely distributed and dominant *Gentiana* species in the THR. Natural hybridization has been confirmed between *G. straminea* and *G. siphonantha* in two studies (Li *et al.* 2008; Hu *et al.* 2016). Hybridization has also been suggested between another two *Gentiana* species (Fu *et al.* 2020). The overlapping flowering time of *Gentiana*, which mostly flower August to September (Ho and Liu 2001), together with shared generalist pollinators such as bumblebees (Duan *et al.* 2007; Liu *et al.* 2014a and references therein), means there are likely to be few pre-pollination reproductive isolating barriers. Despite limited study of hybridization in *Gentiana*, there is a growing body of evidence that hybridization may be common in this alpine genus. A previous study has reported expansion after historical hybridization from a refugium in another *Gentiana* taxa (Fu *et al.* 2020). Hybridization is also detected among some European gentians, for example *G. punctata* and *G. purpurea*, *G. lutea* and *G. purpurea*, from records of specimen collections in E or by Rich and McVeigh (2019). Although the area of hybridization overlaps the potential refugium of *G. straminea* (Lu *et al.* 2015), hybridization between *G. hoae* and *G. straminea* appears to be recent. Additionally, recombinant sequences of nrITS were identified in the five hybrid individuals (Table 4; **see**  **Supporting Information—Appendix S2**). These recombinant sequences could be the result of natural recombination between parental copies. However, cross-hybridization and mispriming during PCR amplification, which could also produce artificial recombinant sequences (Cronn *et al.* 2002), cannot be excluded here, but seem unlikely given the uniform amplification and the presence of ITS-additivity only in these co-occurring populations.

Hybridization is a common phenomenon in plants (Abbott *et al.* 2013), especially on the QTP where a shared pool of a few insect species such as bumblebees take part in pollination of many plant species (Liu *et al.* 2014a and references therein). Hybridization is likely to be an important mechanism underlying speciation in the THR (Wen *et al.* 2014). However, hybrids with clear intermediate morphological characters may represent only a minority of natural hybrid genotypes, and backcross hybrids and introgressed individuals are likely to be overlooked based on morphology alone. More generally, the importance of cryptic biodiversity that results from interspecific hybridization has been largely neglected in previous studies of the THR (though see Hu *et al.* 2016). With genetic analysis, hybridization, even without distinguishable morphological consequences, has been detected in the THR (e.g. Yang *et al.* 2012; Wu *et al.* 2016; Yan *et al.* 2017; Ma *et al.* 2019; Fu *et al.* 2020). As the hybrids we detected are likely to be early generation hybrids such as F1s, further studies of the outcomes of natural hybridization and the maintenance of species boundaries (e.g. Twyford *et al.* 2015), as well as the fitness of hybrids relative to their parents, should be conducted to provide a more precise understanding of the postzygotic barriers at play in *Gentiana* (Abbott and Brennan 2014).

## Taxonomic description of *Gentiana hoae*

*Gentiana hoae* P.C. Fu & S.L. Chen, sp. nov.—Holotype: CHINA. Qinghai Province, ca. 40 km SW of Yushu, 12 August 2017, 97°12′04″E, 32°46′40″N. Fu2017046.

Perennials 6–20 cm tall. Roots to 15 cm. Stems ascending, slender, glabrous. Basal leaves petiole 0.5–2 cm, membranous; leaf blade lanceolate to linear-lanceolate, 3–10 cm × 4–10 mm, margin scabrous, base narrowed, apex acuminate, veins 1–3. Stem leaves three or four pairs; petiole 5–10 mm; leaf blade lanceolate to linear-lanceolate, 1.0–3.0 cm × 2–4 mm, apex acuminate, margin scabrous, mid-vein distinct. Flowers solitary, rarely in cymes. Pedicel purple, to 4.5 cm. Calyx tube narrowly obconic, 1.0–1.4 cm, membranous, margin entire; lobes 5, narrowly elliptic to linear, subequal, 3–8 mm, herbaceous, base not narrowed, margin scabrous, apex acute, mid-vein distinct. Corolla inside pale blue, outside dark brown, tubular to funnelform, 2–3 cm; lobes triangular-elliptic, 4–6 mm, margin entire, apex acute rounded; plicae narrowly triangular, 2–2.5 mm, margin denticulate, apex acute. Stamens inserted just below middle of corolla tube; filaments 5–8 mm; anthers narrowly ellipsoid, 1.5–2 mm. Style 1–2 mm; stigma lobes oblong. Capsules sessile, ovoid-ellipsoid, 1.2–1.5 cm. Seeds brown, ellipsoid, 1.4–1.6 mm. Fl. and fr. August–September.

The new species differs from all other species of *Gentiana* section *Cruciata*, except for *G. lhassica*, in not split calyx tube and solitary flowers. It differs from the latter species in narrower basal leaf balde, stem leaves, calyx and corolla lobes, and lighter corolla colour.

*Distribution*.—The new species has been found in Southwest Qinghai (Yushu), Northeast Tibet (Changdu) and west border of Sichuan (Litang).

*Paratypes*.—Qinghai Province, Nangqian, August 1972 (HNWP, 28499); Qinghai Province, Nangqian, Xuebayaela Mountain, August 2017, N. Fu2017072; Tibet, Chuangdu, Tuoba, August 2017, N. Fu2017135.

*Ecology*.—*Gentiana hoae* grows in alpine meadow or shrubs, usually on sunny slopes. Its habitat is very similar with *G. lhassica*, and the ecological differences between these two species are currently unclear.

*Etymology*.—The pecies epithet is chosen in honour of Prof. Ting-Nong Ho for her systematic work in the taxonomy of Gentianaceae (Ho and Liu 2001, 2015).

*Conservation status*.—The investigated populations generally have more than 1000 individuals, and new species should be regarded as ‘Least Concern’ based on the IUCN (2012) criteria (https://portals.iucn.org/library/node/10315).

## Supporting Information

The following additional information is available in the online version of this article—

Appendix S1. Nucleotide variation of plastid haplotypes identified in populations of *Gentiana hoae* and its closely related species. Dots represent the first nucleotide(s) in each column.

Appendix S2. Nucleotide variation of the internal transcribed spacer regions of the nuclear ribosomal ribotypes identified in populations of *Gentiana hoae* and its closely related species. Dots represent the first nucleotide(s) in each column.

Appendix S3. Phylogenetic relationship of the internal transcribed spacer regions of the nuclear ribosomal (nrITS) ribotypes from populations of *Gentiana hoae* and *G. straminea*.

Appendix S4. Phylogenetic relationship of plastid (*trnS*-*trnG* and *rpl32-trnL* loci) haplotypes from populations of *Gentiana hoae* and *G. straminea*.

plaa068_suppl_Supplementary_Material

## Data Availability

All data are provided within the text, tables, figures and supplementary.

## Notes

**Evolution & Diversity. Chief Editor:** Jeremy Beaulieu
