# Supplementary material for: Recurrent hybridization underlies the evolution of novelty in Gentiana (Gentianaceae) in the Qinghai-Tibetan Plateau
Source: AoB Plants. 2020 Dec 2;13(1):plaa068. doi: 10.1093/aobpla/plaa068 (PMC7821390; doi:10.1093/aobpla/plaa068)
Supplement: plaa068_suppl_Supplementary_Material [file plaa068_suppl_supplementary_material.pdf]

Appendix S1. Nucleotide variation of plastid haplotypes identified in populations of *Gentiana hoae* and its closely related species. Dots represent the first nucleotide(s) in each column.

Hc1	C	T	A	A	-	A	A	G	C	C	ATTTT	T	-	-	T	TCTATGTGTACATATCCATATAATA
Hc2	.	.	.	-	.	.	.	.	.	.	.	.	T	.	.	.
Hc3	.	.	.	.	.	.	.	.	.	.	.	.	T	.	.	.
Hc4	A	.	.	.	.	.	.	.	.	.	.	.	.	.	.	.
Hc5	.	.	.	.	.	.	.	.	.	.	.	-	.	.	.	.
Hc6	.	.	.	.	.	.	.	.	.	A	.	.	.	.	.	.
Hc7	.	.	.	.	.	.	.	.	.	A	.	.	T	.	.	.
Hc8	.	.	.	.	A	.	C	.	.	.	.	.	.	.	.	.
Hc9	.	.	.	.	A	.	C	.	.	A	.	.	.	.	.	.
Hc10	.	.	.	-	.	.	.	.	.	.	.	.	.	.	.	.
Hc11	.	.	.	.	.	.	.	.	.	.	.	.	T	T	.	.
Hc12	.	.	.	-	.	.	.	.	.	.	.	.	T	.	A	.
Hc13	.	.	.	.	.	.	.	.	.	.	.	.	T	T	A	.
Hc14	.	.	.	-	.	.	.	.	.	A	.	.	T	.	.	.
Hc15	.	.	.	.	A	.	C	.	.	A	.	.	T	.	.	.
Hc16	.	.	.	.	A	.	C	.	.	.	.	-	.	.	.	.
Hc17	.	C	T	-	.	G	C	A	-	A	-----	-	.	.	.	-------------------------

Appendix S2. Nucleotide variation of the internal transcribed spacer regions of the nuclear ribosomal ribotypes identified in populations of *Gentiana hoae* and its closely related species. Dots represent the first nucleotide(s) in each column.

Sample	Name	Nucleotides																								
		1	2	3	4	5	6	7	8	9	10	11	12	13	14	15	16	17	18	19	20	21	22	23	24	25
G. holis	H1	G	C	A	T	C	G	C	C	A	A	C	C	C	C	---	G	G	G	A	T	C	T	G	G	G
	H2	.	.	.	.	.	.	.	.	.	.	.	.	.	.	.	.	.	.	-	.	.	.	.	.	.
	H3	.	.	.	.	.	.	.	.	.	.	.	.	.	.	.	.	.	.	.	.	.	.	.	.	.
	H4	.	.	.	.	.	.	.	.	.	.	.	.	.	.	.	.	.	.	.	.	.	.	.	.	.
	H5	.	.	C	.	.	.	.	.	.	.	T	.	.	.	.	.	.	.	.	.	.	.	.	.	.
	H6	.	.	C	.	.	.	.	.	.	.	T	.	.	.	.	.	.	.	.	.	.	.	.	.	.
	H7	.	.	C	.	.	.	.	.	.	.	T	.	.	.	.	.	.	.	.	.	T	.	.	.	.
	H8	.	.	C	.	.	.	.	.	.	.	T	.	.	.	.	.	.	.	.	.	.	.	.	.	.
	H9	.	.	C	.	.	.	.	.	.	.	.	.	.	.	.	.	.	.	.	.	.	.	A	.	.
	H10	.	.	.	.	.	.	.	.	.	.	.	.	.	.	.	.	.	.	.	.	.	.	A	.	.
	H11	.	.	.	.	.	.	.	.	.	.	.	.	.	.	.	.	.	.	.	.	.	.	.	.	.
	H12	.	.	.	.	.	.	.	.	.	.	.	.	.	.	.	.	.	.	.	.	.	.	A	.	.
G. straminea	S1	.	.	.	.	A	.	.	.	.	.	.	.	.	T	TGA	.	.	.	.	.	.	G	.	.	.
	S2	.	.	.	.	A	.	.	.	.	.	.	.	.	T	TGA	.	.	.	.	.	.	G	.	.	.
	S3	.	.	.	.	A	.	.	T	.	.	.	.	.	T	TGA	.	.	.	.	.	.	G	.	.	.
	S4	.	.	.	.	A	.	.	T	.	.	.	.	.	T	TGA	.	.	.	.	.	.	G	.	T	.
	S5	.	.	.	.	A	.	.	T	.	.	.	.	.	T	TGA	.	.	.	.	.	.	G	.	.	.
	S6	.	.	.	.	A	.	.	.	.	.	.	.	.	T	TGA	.	.	.	.	.	.	G	.	.	.
	S7	.	.	.	.	A	.	.	.	.	.	.	.	.	T	TGA	.	.	.	.	.	.	G	.	.	.

	S8	.	.	.	.	A	.	.	.	.	.	.	T	.	T	TGA	.	.	.	.	.	.	G	.	.	.
	H3	.	.	.	.	.	.	.	.	.	.	.	.	.	.	.	.	.	.	.	.	.	.	.	.	.
	R1	.	.	.	.	.	.	.	.	.	.	.	.	.	.	.	.	.	.	.	.	.	G	.	.	.
Hyb1	R2	.	.	.	.	.	.	.	.	.	.	.	.	T	.	.	.	.	.	.	.	.	.	.	.	.
	R3	.	.	.	.	.	.	.	.	C	.	.	.	.	.	.	.	.	.	.	.	.	.	.	.	.
	R4	.	.	.	.	A	.	.	.	.	.	.	.	.	T	TGA	.	.	.	.	.	.	G	.	.	.
	H3	.	.	.	.	.	.	.	.	.	.	.	.	.	.	.	.	.	.	.	.	.	.	.	.	.
	R5	.	.	G	.	.	.	.	.	.	.	.	.	.	.	.	.	.	.	.	.	.	.	.	.	.
Hyb2_1	R6	A	.	.	.	.	.	T	.	.	.	.	.	.	.	.	.	.	A	.	.	.	.	.	.	.
	R7	.	.	.	.	A	.	.	.	.	.	.	.	.	T	TGA	.	.	.	.	.	.	G	.	.	.
	R8	.	.	.	.	A	.	.	.	.	.	.	.	.	T	TGA	.	.	.	.	.	.	.	.	.	.
	R9	.	.	.	.	A	.	.	.	.	.	.	.	.	T	TGA	.	.	.	.	.	.	.	.	.	.
	H3	.	.	.	.	.	.	.	.	.	.	.	.	.	.	.	.	.	.	.	.	.	.	.	.	.
	R10	.	.	.	.	.	.	.	.	.	.	.	.	.	.	.	.	.	.	.	.	.	.	.	.	.
Hyb2_2	R11	.	.	.	.	.	A	.	.	.	.	.	.	.	.	.	A	A	.	.	.	.	G	.	.	.
	S1	.	.	.	.	A	.	.	.	.	.	.	.	.	T	TGA	.	.	.	.	.	.	G	.	.	.
	R12	.	.	.	.	A	.	.	.	.	.	.	.	.	T	TGA	.	.	.	.	.	.	G	.	.	.
	R13	.	A	.	C	.	.	.	A	.	G	.	.	.	.	.	.	.	.	.	.	.	.	.	.	.
	R14	.	.	.	.	.	.	.	.	.	.	.	.	.	.	.	.	.	.	.	.	.	.	.	.	.
	R15	.	.	.	.	.	.	.	.	.	.	.	.	.	.	.	.	.	.	.	.	.	.	.	.	.
Hyb2_3	R16	.	.	.	.	.	.	.	.	.	.	.	.	.	.	.	.	.	.	.	.	.	G	.	.	A
	R17	.	.	.	.	.	.	.	.	.	.	.	.	.	.	.	.	.	.	.	.	.	.	.	.	.
	R18	.	.	.	.	.	.	.	.	.	.	.	.	.	.	.	.	.	.	.	.	.	.	.	.	.
	R19	.	.	.	.	A	.	.	.	.	.	.	.	.	.	.	.	.	.	.	.	.	.	.	.	.
	R20	.	.	.	.	A	.	.	.	.	.	.	.	.	T	TGA	.	.	.	.	.	.	G	.	.	.

	S1	.	.	.	.	A	.	.	.	.	.	.	.	.	T	TGA	.	.	.	.	.	.	G	.	.	.
Hyb3	R21	.	.	.	.	.	.	.	.	.	.	.	.	.	.	.	.	.	.	.	.	T	.	.	.	.
	R22	.	.	.	.	.	.	.	.	.	.	.	.	.	.	.	.	.	.	.	C	C	G	.	.	.
	S1	.	.	.	.	A	.	.	.	.	.	.	.	.	T	TGA	.	.	.	.	.	.	G	.	.	.
	R23	.	.	.	.	A	.	.	.	.	.	.	.	.	T	TGA	.	.	.	.	.	.	G	.	.	.
	R24	.	.	.	.	A	.	.	.	.	.	.	.	.	T	TGA	.	.	.	.	.	.	G	.	.	.
	R25	.	.	.	.	A	.	.	.	.	.	.	.	.	T	TGA	.	.	.	.	.	.	G	.	.	.
G. lhassica	L1	.	.	.	.	.	.	.	.	.	.	.	.	.	.	.	.	.	.	.	.	.	.	.	.	.

(continued)

Sample	Name	Nucleotides																						
		26	27	28	29	30	31	32	33	34	35	36	37	38	39	40	41	42	43	44	45	46	47	48	49
G. holis	H1	C	C	G	G	T	C	C	C	T	T	G	T	G	A	C	A	T	T	C	T	C	A	C	C
	H2	.	.	.	.	.	.	-	.	.	.	.	.	.	.	.	.	.	.	.	.	.	.	.	.
	H3	.	.	.	.	.	.	-	.	.	.	.	.	.	.	.	.	.	.	.	.	.	.	.	.
	H4	.	.	.	.	.	.	-	.	.	.	.	.	.	.	.	.	.	.	.	.	.	.	T	.
	H5	.	T	.	.	.	.	-	.	.	.	.	.	.	.	.	.	.	A	.	.	.	.	.	.
	H6	T	T	.	.	.	.	-	.	.	.	.	.	.	.	.	.	.	A	.	.	.	.	.	.
	H7	.	T	.	.	.	.	-	.	.	.	.	.	.	.	.	.	.	.	.	.	.	.	.	.
	H8	.	T	.	.	.	.	-	.	.	.	.	.	.	.	.	.	.	.	.	.	.	.	.	.
	H9	.	T	.	.	.	.	-	.	.	.	.	.	.	.	.	.	.	.	.	.	.	.	.	.
	H10	.	T	.	.	.	.	-	.	.	.	.	.	.	.	.	.	.	.	.	.	.	.	.	.
	H11	.	T	.	.	.	.	-	.	.	.	.	.	.	.	.	.	.	.	.	.	.	.	.	.
	H12	.	.	.	.	.	.	-	.	.	.	.	.	.	.	.	.	.	.	.	.	.	.	.	.

	S1	.	.	.	.	.	-	-	T	.	.	.	.	.	.	.	.	.	.	.	.	.	.	.	.
	S2	.	.	.	.	.	-	-	T	.	.	.	.	.	.	.	.	.	.	T	.	.	.	.	.
	S3	.	.	.	.	.	-	-	T	.	.	.	.	.	.	.	.	.	.	.	.	.	.	.	.
G. straminea	S4	.	.	.	.	.	-	-	T	.	.	.	.	.	.	.	.	.	.	.	.	.	.	.	.
	S5	.	.	.	.	.	-	-	T	.	.	.	.	.	.	.	.	.	.	T	.	.	.	.	.
	S6	.	.	.	.	.	-	-	T	.	.	.	.	.	.	T	.	.	.	.	.	.	.	.	.
	S7	.	.	.	.	.	-	-	T	.	.	.	.	.	.	.	G	.	.	.	.	.	.	.	.
	S8	.	.	.	.	.	-	-	T	.	.	.	.	.	.	.	.	.	.	.	.	.	.	.	.
	H3	.	.	.	.	.	.	-	.	.	.	.	.	.	.	.	.	.	.	.	.	.	.	.	.
	R1	.	.	.	.	.	.	-	.	.	C	.	.	.	.	.	.	.	.	.	.	.	.	.	.
Hyb1	R2	.	.	.	.	.	-	-	T	.	C	.	.	.	.	.	.	.	.	.	.	.	.	.	.
	R3	.	.	.	.	.	-	-	T	.	C	.	.	.	.	.	.	.	.	.	.	.	.	.	.
	R4	.	.	.	.	.	-	-	T	.	C	.	.	.	G	.	.	.	.	.	.	.	.	.	.
	H3	.	.	.	.	.	.	-	.	.	.	.	.	.	.	.	.	.	.	.	.	.	.	.	.
	R5	.	.	.	.	.	.	-	.	.	.	.	.	.	.	.	.	.	.	.	.	.	.	.	.
Hyb2_1	R6	.	.	.	.	.	-	-	T	.	C	.	.	.	.	.	.	.	.	.	.	.	.	.	.
	R7	.	.	.	.	.	-	-	T	.	C	.	.	.	.	.	.	.	.	.	.	.	.	.	.
	R8	.	.	.	.	.	.	-	.	.	.	.	.	.	.	.	.	C	.	.	.	T	.	.	.
	R9	.	.	.	.	.	.	-	.	.	.	.	.	.	.	.	.	.	.	.	.	.	.	.	.
	H3	.	.	.	.	.	.	-	.	.	.	.	.	.	.	.	.	.	.	.	.	.	.	.	.
	R10	.	.	.	.	.	.	-	.	.	.	.	.	A	.	.	.	.	.	.	.	.	.	.	.
Hyb2_2	R11	.	.	.	.	.	.	-	.	.	.	.	.	.	.	.	.	.	.	.	.	.	.	.	T
	S1	.	.	.	.	.	-	-	T	.	.	.	.	.	.	.	.	.	.	.	.	.	.	.	.
	R12	.	.	.	.	.	.	-	.	.	.	.	.	.	.	.	.	.	.	.	.	.	.	.	.
Hyb2_3	R13	.	.	.	.	.	.	-	.	.	.	.	.	.	.	.	.	.	.	.	.	.	C	.	.

	R14	.	.	.	.	.	-	-	T	.	.	.	C	.	.	.	.	.	.	.	.	.	.	.	.
	R15	.	.	.	.	.	.	-	.	.	.	.	C	.	.	.	.	.	.	.	.	.	.	.	.
	R16	.	.	.	.	.	-	-	T	.	.	.	.	.	.	.	.	.	.	.	.	.	.	.	.
	R17	.	.	.	.	.	-	-	T	C	.	.	.	.	.	.	.	.	.	.	.	.	.	.	.
	R18	.	.	.	.	.	-	-	T	.	.	.	.	.	.	.	.	.	.	.	.	.	.	.	.
	R19	.	.	.	.	.	-	-	T	.	.	.	.	.	.	.	.	.	.	.	.	.	.	.	.
	R20	.	.	A	.	.	-	-	T	.	.	.	.	.	.	.	.	.	.	.	.	.	.	.	.
	S1	.	.	.	.	.	-	-	T	.	.	.	.	.	.	.	.	.	.	.	.	.	.	.	.
	R21	T	.	.	.	.	.	-	.	.	.	.	.	.	.	.	.	.	A	.	.	.	.	.	.
	R22	.	.	.	.	C	-	-	T	.	.	.	.	.	.	.	.	.	A	.	.	.	.	.	.
Hyb3	S1	.	.	.	.	.	-	-	T	.	.	.	.	.	.	.	.	.	.	.	.	.	.	.	.
	R23	.	.	.	.	.	.	-	.	.	.	.	.	.	.	.	.	.	A	.	C	.	.	.	.
	R24	.	.	.	T	.	.	-	.	.	.	.	.	.	.	.	.	.	A	.	.	.	.	.	.
	R25	.	.	.	.	.	.	-	.	.	.	.	.	.	.	.	.	.	A	.	.	.	.	.	.
G. lhassica	L1	.	.	.	.	.	.	-	.	.	.	A	.	.	.	.	.	.	.	.	.	.	.	.	.

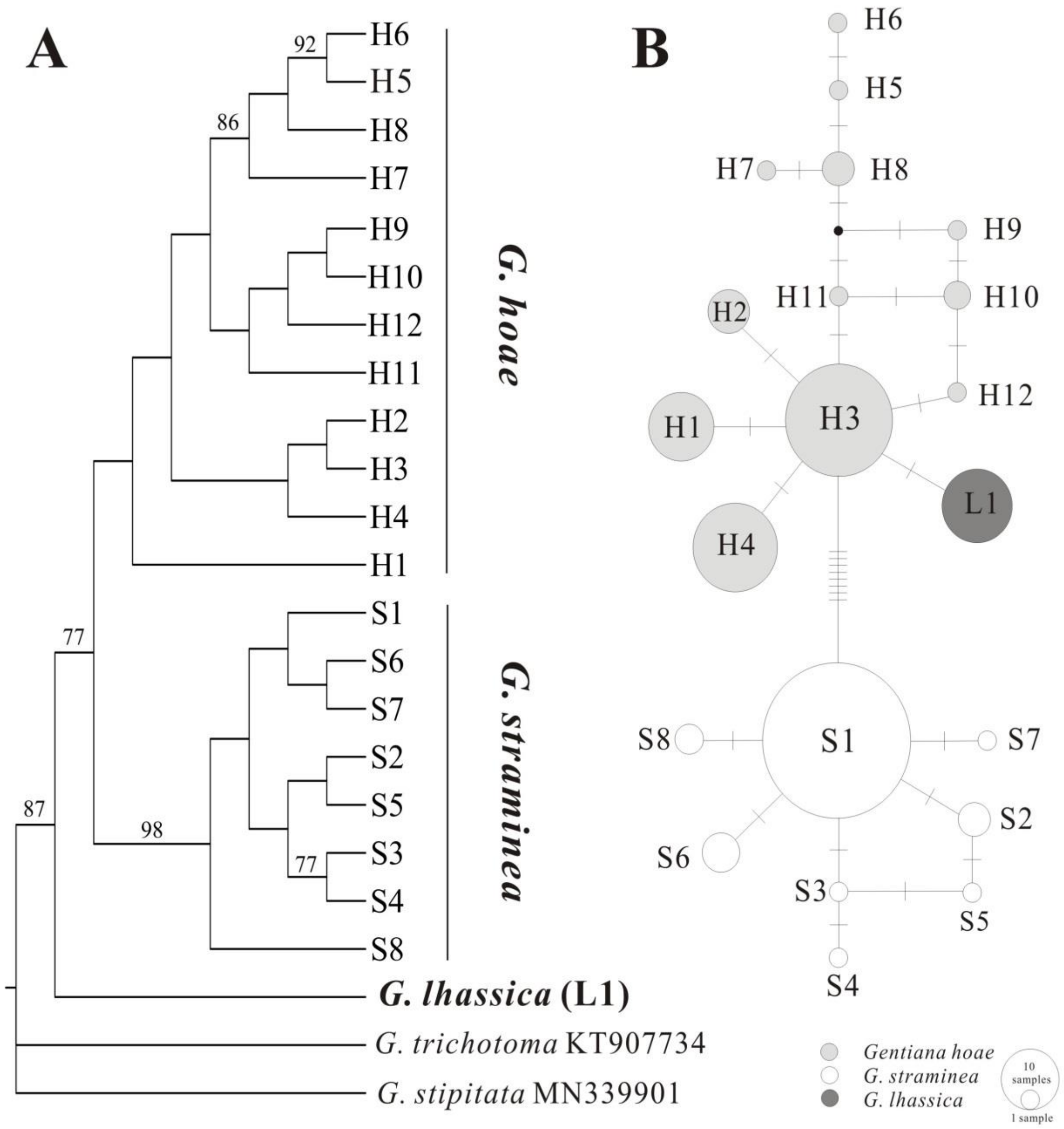

Appendix S3. Phylogenetic relationship of the internal transcribed spacer regions of the nuclear ribosomal (nrITS) ribotypes from populations of *Gentiana hoae* and *G. straminea*. A, ML tree. Bootstrap support values obtained from maximum likelihood analyses are presented above branches (values less than 60% are not showed). B, network of the nrITS ribotypes. The relative sizes of the circles in the network are proportional to ribotype frequencies. One short dash represents one nucleotide variation and black dots represent missing ribotypes.

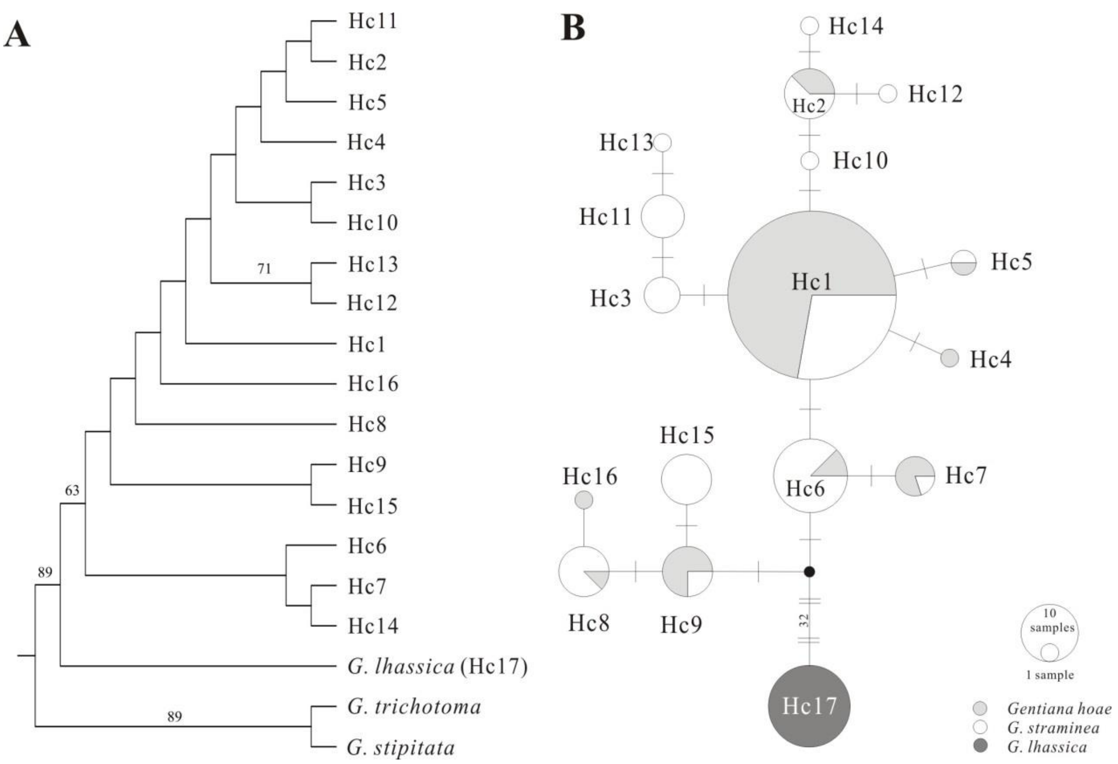

Appendix S4. Phylogenetic relationship of plastid (*trnS-trnG* and *rpl32-trnL* loci) haplotypes from populations of *Gentiana hoae* and *G. straminea*. A, ML tree. Bootstrap support values obtained from maximum likelihood analyses are presented above branches (values less than 60% are not showed). B, network of the plastid haplotypes. The relative sizes of the circles in the network are proportional to haplotype frequencies. One short dash represents one nucleotide variation and black dots represent missing haplotypes.
